# Evaluation of dietary *Perilla frutescens* seed on performance and carcass quality in finishing castrated male Songliao black pigs

**DOI:** 10.1002/vms3.690

**Published:** 2022-01-11

**Authors:** Ji Qiao Xia, XinMiao He, Lan Wang, Liang Wang, Dong Ji Zhang, Ji Feng Wang, Di Liu

**Affiliations:** ^1^ College of Animal Science and Technology Institute of Animal Nutrition Northeast Agricultural University Harbin China; ^2^ Key Laboratory of Combining Farming and Animal Husbandry Ministry of Agriculture Heilongjiang Academy of Agricultural Sciences Animal Husbandry Research Institute Harbin China; ^3^ Laboratory of Animal Husbandry and Veterinary Daxing'anling Academy of Agriculture and Forestry Sciences Daxing'anling Heilongjiang China

**Keywords:** growth performance, meat nutritional parameters, meat quality, *Perilla frutescens* seed, Songliao black pig

## Abstract

**Objectives:**

This study was conducted to investigate the effects of dietary supplementation of *Perilla frutescens* seed (PFS) on growth performance, blood profiles, meat quality and meat nutrient characteristics in finishing castrated male Songliao black pigs.

**Methods:**

A total of 80 castrated male Songliao black pigs with an average initial body weight (BW) of 84.1 ± 2.1 kg were used in a 75 days feeding trial. All pigs were randomly assigned into four dietary treatments: CON, basal diet; PFS3.0, basal diet + 3.0% of PFS; PFS6.0, basal diet + 6.0% of PFS and PFS9.0, basal diet + 9.0% of PFS.

**Results:**

As a result of this experiment, dietary supplementation of PFS improved the growth performance parameters, blood albumin and blood lipid parameters. Whereas, on FBW, average daily feed intake and average daily gain there showed a non‐dose‐dependent manner that pigs in PFS9.0 had lowest performance compared with other two PFS treatments. Furthermore, meat colour of yellowness, pH, cook meat rate, moisture, crude protein and crude fat were increased by PFS addition. However, lower growth performance was observed in PFS9.0 group. As well as, dietary inclusion of PFS also alters the meat amino acid composition and meat fatty acids composition. Particularly, umami amino acid contents and polyunsaturated fatty acid were all enhanced by PFS addition.

**Conclusions:**

In summary, dietary supplementation of PFS have beneficial effects on the performance and meat quality and nutritional values in Songliao black pigs.

## Graphical abstract

The current study evaluated the effects of PF seed as a dietary ingredient to reduce the use of soybean meal on performance and meat characteristics in finishing male Songliao black pigs. The results of this study showed that PF seed positively influenced the growth and meat quality parameters in Songliao black pigs, which provides evidence and insight for the application of PF seed in the swine industry.

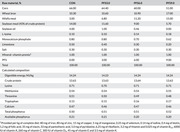

## 1 INTRODUCTION

It has been widely accepted that soybean meal (SBM) is a primary source of dietary protein and plays an important role in animal feed (Sun et al., 2020). Due to agricultural geography, seasonal differences and insufficient production, it is difficult to meet the requirements of the rapid growth of the swine industry with SBM (Florou‐Paneri et al., 2014). Thus, identification of other ingredients to replace or reduce the use of SBM as a feedstuff is necessary. Moreover, the demand for safe and quality pork is increasing, requiring not only increased quantity but also on the nutritional.

*Perilla frutescens* (PF) is an annual herbal plant that belongs to the family of mint, Lamiaceae (Zhou et al., 2014). China is considered the primary gene centre for PF although it is also widely distributed in many other Asian countries, such as Japan, South Korea, Vietnam and India, where it is known by different local names (Yu et al., 2017). PF has been traditionally used as a medicinal herb for centuries to alleviate many different conditions, such as cancer, colds, fever, headache and various intestinal disorders (Ha et al., 2012; Yang et al., 2012). Currently, it is considered an industrial crop with both medicinal and dietary value; the stem, leaf and seed are all commonly used (Zhao et al., 2019). In recent years, interest from the livestock industry has focused on PF seed (PFS) and its by‐products due to the high protein, amino acid (AA), fatty acid (FA) and vitamin content (Wang et al., 2020). PFS products have received highly positive evaluation in livestock animals. For instance, Song et al. (2014) reported that in broilers, PFS extracts significantly improved immune functions such as T lymphocyte rate, serum IgG concentration and the immune organ index. Wang and Sun (2018) indicated that dietary supplementation of PFS extracts increased the finial body weight (FBW) and average daily gain while decreasing the feed conversion ratio (FCR) in finishing pigs.

Furthermore, the supplementation of PFS products also resulted in positive influences in animal product quality. Peng et al. (2018) reviewed the application of PFS products in poultry and reported that PFS products not only improved the meat quality of broilers, but they also positively affected the egg production ratio and egg FA content in laying hens. Similar results were also shown in pigs, as the addition of PFS extracts increased water‐holding ratio, loin muscle area, intramuscular fat content and meat FA composition (Hu et al., 2011; Zhou et al., 2019).

The Songliao black pig was a native meat breeding swine in northern China to exhibit advantages in growth rate and lean meat percentage (Hui, 2017). It is hypothesised that dietary supplementation of PFS may have a positive influence on the meat quality, flavour and nutritional parameters in Songliao black pig meat. Therefore, the objective of this experiment was to evaluate the effects of dietary inclusion of PFS on growth performance, blood profiles, meat quality and meat nutritional parameters in finishing castrated male Songliao black pigs.

## 2 MATERIALS AND METHODS

The experimental protocols used in this study were reviewed and approved by the Animal Care and Use Committee of Northeast Agricultural University, People's Republic of China. The experiment was carried out at the Experimental Base of Animal Husbandry and Veterinary Department, Academy of Agriculture and Forestry Sciences at Daxing'anling, China. The experiment lasted 75 days from July to September 2019.

### 2.1 Experimental design, animals, diets and housing

A total of 80 castrated, male, healthy, Songliao black pigs with an average initial body weight (BW) of 84.1 ± 2.1 kg were used in this feeding trial. Pigs were randomly allotted to one of four dietary treatments according to initial BW in a randomised block design. There were 20 replicated pens per treatment with 1 pig per pen. Dietary treatments were: (1) CON (basal diet), (2) PFS3.0 (basal diet + 3.0% of PFS), (3) PFS6.0 (basal diet + 6.0% of PFS) and (4) PFS9.0 (basal diet + 9.0% of PFS). All of the diets were formulated to meet or exceed the NRC (2012) requirement for finishing pigs and the China Feeding Standard of Swine (2004) and were presented in mash form (Table 1). The PFS replaced the same content of other dietary compositions. All of the pigs were housed in an environmentally controlled room with a slatted plastic floor. Each pen was provided with a one‐side self‐feeder and a nipple drinker to allow ad libitum access to feed and water during the experiment. Target room temperature and humidity were 25°C and 60%, respectively.

**TABLE 1:** Basal diet composition (as‐fed basis)

Raw material, %	CON	PFS3.0	PFS6.0	PFS9.0
Corn	66.00	64.40	60.00	51.00
Wheat bran	10.30	10.60	10.90	17.00
Alfalfa meal	3.00	6.80	11.20	15.00
Soybean meal (45% of crude protein)	14.00	11.60	9.00	5.70
Soybean oil	4.00	1.00	0.46	0.00
L‐lysine	0.10	0.10	0.14	0.18
Monocalcium phosphate	0.80	0.80	0.70	0.62
Limestone	0.50	0.40	0.30	0.20
Salt	0.30	0.30	0.30	0.30
Mineral–vitamin premix^1^	1.00	1.00	1.00	1.00
PFS	0.00	3.00	6.00	9.00
Total	100.00	100.00	100.00	100.00
Calculated composition				
Digestible energy, MJ/kg	14.24	14.23	14.24	14.24
Crude protein	13.63	13.63	13.64	13.63
Lysine	0.71	0.70	0.71	0.71
Methionine	0.54	0.54	0.54	0.55
Threonine	0.51	0.50	0.49	0.48
Tryptophan	0.15	0.15	0.16	0.17
Calcium	0.47	0.46	0.47	0.46
Total phosphorus	0.50	0.51	0.51	0.52
Available phosphorus	0.21	0.21	0.20	0.20

^1^ Provided per kg of complete diet: 80 mg of iron, 80 mg of zinc, 15 mg of copper, 5 mg of manganese, 0.25 mg of selenium, 0.14 mg of iodine, 0.4 mg of biotin, 1.2 mg of folic acid, 35 mg of niacin, 33 mg of pantothenic acid, 5.0 mg of pyridoxine, 6.25 mg of riboflavin, 3.2 mg of thiamin and 0.025 mg of vitamin B_12_, 6000 IU of vitamin A, 260 mg of vitamin C, 300 IU of vitamin D_3_, 45 mg of vitamin E and 3.5 mg of vitamin K.

### 2.2 Sampling and measurements

Pigs were weighed initially and at the end of the experiment, and feed consumption was recorded throughout the experiment. Average daily gain (ADG), average daily feed intake (ADFI) and feed conversion ratio (FCR) were then calculated. At the end of the experiment, three pigs were randomly selected from each treatment for acquisition of blood samples. Blood samples were taken from the pigs via jugular venipuncture using K3EDTA vacuum tubes (Becton, Dickinson and Co., Franklin Lakes, NJ, USA). After collection, the serum samples from tubes were centrifuged (3000 × g) for 15 min at 4°C and stored until further analysis. All of the blood profiles, including total protein, albumin, globulin, triacylglycerol, cholesterol, low‐density lipoprotein cholesterol (LDL‐C), high‐density lipoprotein cholesterol (HDL‐C), blood urine nitrogen (BUN) and blood glucose were analysed by using an automatic biochemistry analyser (UniCel DxC 800 SYNCHRON, Beckman Coulter, USA) with commercial kits according to the manufacturer protocols.

At the end of the experiment, 15 pigs per treatment were randomly chosen and slaughtered at a local commercial slaughterhouse (Daxing'anling, China). In brief, the pigs were electrically stunned, bled and eviscerated. Then, the carcass was split longitudinally, and cooling flushing was carried out for 24 h at 4°C. After chilling, longissimus dorsi muscles were taken from the carcasses. Before further evaluation, meat samples were thawed at an ambient temperature. Reflectance‐spectrometry measurements of lightness (L*), redness (a*) and yellowness (b*) were determined with a Chroma Meter CR‐410 chroma meter (Konica Minolta Sensing, Inc., Osaka, Japan). Duplicate pH values of each sample were measured with a pH meter (ORION 3‐Star, Thermo Fisher Scientific, MA, USA) at 45 min and 24 h. Water‐holding capacity (WHC) was measured according to the methods described by previous researchers (Sun & Kim, 2020). Briefly, 1 g sample was pressed at 300 psi for 3 min onto 125‐mm‐diameter filter paper. The areas of the pressed sample and expressed moisture were delineated and determined using a sensor (Digitizing Area Line Sensor, MT‐10S, M.T. Precision Co., Ltd.). The ratio of water area to meat area was calculated, giving a measure for WHC (a smaller ratio indicating a higher WHC). Shear force values of longissimus dorsi muscles in offspring pigs were determined with a CLM‐4 digital explicit muscle tenderness meter (School of Engineering, Northeast Agricultural University). The force record value during shearing was collected, and the average value was calculated. Drip loss was measured using 4 g of meat via the plastic‐bag method described by Honikel (1998). Briefly, 5 g of meat sample was separately heat treated in plastic bags in a water bath (72°C) for 5 min to determine the cook meat rate. Samples were cooled 40 min under watercourse. The meat cook rate was calculated as (sample weight before cooking – sample weight after cooking)/sample weight before cooking × 100. The determination of pressing loss was done according to the method by Farouk and Wieliczko (2003). Briefly, 5 g of meat sample was put under pressure of 35 kg for 5 min and then taken off and weighed. The pressing loss was calculated as (sample weight before pressure – sample weight after pressure)/sample weight before pressure × 100.

The determination of meat chemical composition followed the methods described by Cheng et al. (2017). In brief, crude protein was determined by the Kjeldahl method and crude fat was determined by Soxhlet extraction. Ash refers to the residue remaining after incineration of all of the organic materials in a high temperature furnace at 600°C.

Prior to AA and FA analysis, meat samples were first homogenised. For AA analysis, 80 mg of sample was added into 1000 μl pre‐treatment solution (acetonitrile to water at a 1:1 ratio) and shaken for 1 h. Then, samples were centrifuged at 13,200 rpm/min for 10 min, and the supernatant taken for further analysis. The AA composition in the supernatant was analysed via liquid chromatography‐mass spectrometry (Liquid phase: LC‐20AD, Shimadzu, Japan; Mass Spectrometry: 5500 QTRAP LC‐MS/MS, AB SCIEX, USA). For FA analysis, 100 mg of sample was added to 2 ml of n‐hexane and shaken at 50°C for 0.5 h. At the time of 0.5 h, 3 ml of KOH methanol solution (0.4 mol/L) were added and the sample was shaken for another 0.5 h at 50°C. After 1 h shaking, the samples were mixed with 1 ml of water and 2 ml of n‐hexane. The mixture was then set aside for layering. The upper layer was collected and used for FA determination. The FAs were detected via a gas chromatography‐mass spectrometry (GC‐MS 7890B‐5977A, Agilent, USA). Both AA and FA analysis determination was done following the manufacturer's protocol; the AA and FA contents are expressed as percentages.

### 2.3 Statistical analysis

The data were collected and preliminary processed using Excel. Then, statistical analysis of data was performed using SPSS 17.0 (SPSS, Chicage, IL, USA). One‐way ANOVA was used to determine differences among all diets. Duncan's multiple range test was used to compare the means of different diets. The individual pigs were used as the experimental units. Variability in data was expressed as a pool as means ± standard deviation (SD) and a probability level of less than 0.05 was considered to be statistically significant.

## 3 RESULTS

### 3.1 Growth performance and blood profiles

Growth performance results are shown in Table 2. PFS9.0 observed lowest FBW, ADFI and ADG compared with other treatments; furthermore, there were no significant differences among other dietary treatments. As described in Table 3, PFM6.0 treatment pigs had higher concentrations of blood albumin and BUN than pigs in the CON group. Furthermore, all of three PFM treatments had higher A/G values compared with CON treatment. The CON pigs had higher blood lipid parameters such as triacylglycerol, cholesterol and LDL‐C concentration than pigs fed the PFM3.0 diet.

**TABLE 2:** Effects of dietary supplementation of *Perilla frutescens* seed meal on growth performance in finishing castrated male Songliao black pigs

Items	CON	PFS3.0	PFS6.0	PFS9.0
IBW, kg	84.23 ± 2.21	83.80 ± 1.66	84.33 ± 2.77	84.20 ± 1.91
FBW, kg	130.53 ± 3.68^a^ ^,^ ^b^	132.13 ± 2.75^a^	132.86 ± 2.64^a^	128.80 ± 2.65^b^
ADFI, kg	2.82 ± 0.08^a^	2.83 ± 0.08^a^	2.84 ± 0.07^a^	2.74 ± 0.07^b^
ADG, kg	0.62 ± 0.05^a^ ^,^ ^b^	0.63 ± 0.04^a^	0.64 ± 0.04^a^	0.61 ± 0.04^b^
FCR	4.59 ± 0.31	4.46 ± 0.26	4.43 ± 0.25	4.57 ± 0.21

Abbreviations: ADFI, average daily feed intake; ADG, average daily gain; CON, basal diet; FBW, finial body weight; FCR, feed conversion ratio; IBW, initial body weight; PFS3.0, CON + 3% of *Perilla frutescens* seed; PFS6.0, CON + 6% of *Perilla frutescens* seed; PFS9.0, CON + 9% of *Perilla frutescens* seed.

^a,b^ Means in the same row with different superscripts differ (*p* < 0.05).

**TABLE 3:** Effects of dietary supplementation of *Perilla frutescens* seed meal on blood profiles in finishing castrated male Songliao black pigs

Items	CON	PFS3.0	PFS6.0	PFS9.0
Total protein	72.00 ± 1.69	71.58 ± 3.60	72.64 ± 2.32	72.52 ± 1.30
Albumin	43.18 ± 1.26^b^	44.32 ± 1.55^a^,^b^	45.38 ± 1.70^a^	44.73 ± 1.29^a^ ^,^ ^b^
Globulin	28.82 ± 0.93	27.25 ± 2.28	27.26 ± 1.09	27.80 ± 0.93
A/G	1.50 ± 0.06^b^	1.63 ± 0.10^a^	1.67 ± 0.07^a^	1.61 ± 0.08^a^
Triacylglycerol	0.68 ± 0.16^a^	0.42 ± 0.04^b^	0.59 ± 0.15^a^ ^,^ ^b^	0.51 ± 0.11^a^ ^,^ ^b^
Cholesterol	2.87 ± 0.53^a^	2.11 ± 0.14^b^	2.56 ± 0.30^a^ ^,^ ^b^	2.33 ± 0.66^a^ ^,^ ^b^
LDL‐C	1.44 ± 0.18^a^	1.17 ± 0.15^b^	1.29 ± 0.15^a^ ^,^ ^b^	1.34 ± 0.17^a^ ^,^ ^b^
HDL‐C	0.95 ± 0.14	0.87 ± 0.13	0.99 ± 0.11	1.01 ± 0.19
BUN	5.12 ± 0.55^b^	5.60 ± 0.47^a^ ^,^ ^b^	6.02 ± 0.57^a^	5.28 ± 0.58^a^ ^,^ ^b^
Blood glucose	5.30 ± 1.04^a^	4.73 ± 0.61^b^	5.20 ± 0.84^a^ ^,^ ^b^	5.25 ± 0.78^a^ ^,^ ^b^

Abbreviations: A/G, albumin globulin ratio; BUN, blood urine nitrogen; CON, basal diet; HDL‐C, high‐density lipoprotein cholesterol; LDL‐C, low‐density lipoprotein cholesterol; PFS3.0, CON + 3% of *Perilla frutescens* seed; PFS6.0, CON + 6% of *Perilla frutescens* seed; PFS9.0, CON + 9% of *Perilla frutescens* seed.

^a,b^ Means in the same row with different superscripts differ (*p* < 0.05).

### 3.2 Meat quality and meat chemical composition

The meat quality parameters of pigs fed PFS are shown in Table 4. The meat quality values of lightness and redness were not affected by PFS supplementation, whereas the meat in the PFS9.0 group had the lowest yellowness value compared to the other three treatments. Meat from the CON treatment had the highest of pH value at 45 min and pressing loss percentage compared to the three PFS groups. Furthermore, the CON group also had higher pH value on 24 h compared with the PFS6.0 and PFM9.0 groups. However, the meat cook rate in the CON meat was the lowest among all of the treatments. There were no significant influences on WHC, drip loss at 24 h and shear force among the dietary treatments. As described in Table 5, there were no significant effects of the dietary supplementation of PFS on meat ash among the different treatments. However, the moisture and crude protein were observed significantly higher in all of the PFM groups compared with the CON treatment. Moreover, crude fat in the PFS6.0 diet group was significantly higher than in the CON group.

**TABLE 4:** Effects of dietary supplementation of *Perilla frutescens* seed meal on meat quality in finishing castrated male Songliao black pigs

Items	CON	PFS3.0	PFS6.0	PFS9.0
L*, lightness	52.67 ± 1.70	53.04 ± 1.80	53.79 ± 1.24	53.33 ± 1.14
a*, redness	11.45 ± 1.81	10.53 ± 1.73	10.14 ± 1.33	9.40 ± 1.18
b*, yellowness	8.68 ± 1.33^a^	7.86 ± 0.88^a^	7.63 ± 1.31^a^	5.97 ± 0.97^b^
pH_45min_	6.52 ± 0.05^a^	6.44 ± 0.05^b^	6.42 ± 0.02^b^	6.39 ± 0.03^b^
pH_24h_	5.69 ± 0.06^a^	5.63 ± 0.07^a^ ^,^ ^b^	5.59 ± 0.08^b^	5.57 ± 0.07^b^
Pressing loss %	43.05 ± 1.62^a^	41.02 ± 1.18^b^ ^,^ ^c^	40.48 ± 1.34^b^ ^,^ ^c^	38.98 ± 0.97^c^
WHC, %	41.68 ± 2.90	43.35 ± 1.77	43.14 ± 4.11	44.18 ± 3.29
Drip loss_24h_, %	2.45 ± 0.68	2.28 ± 0.85	2.00 ± 0.20	1.86 ± 0.29
Shear force, N	40.77 ± 1.77	40.64 ± 2.64	38.46 ± 3.00	39.78 ± 2.47
Cook meat rate, %	81.49 ± 1.89^b^	83.75 ± 0.44^a^	84.76 ± 1.75^a^	83.73 ± 1.80^a^

Abbreviations: CON, basal diet; PFS3.0, CON + 3% of *Perilla frutescens* seed; PFS6.0, CON + 6% of *Perilla frutescens* seed; PFS9.0, CON + 9% of *Perilla frutescens* seed; WHC, water‐holding capacity.

^a,b,c^ Means in the same row with different superscripts differ (*p* < 0.05).

**TABLE 5:** Effects of dietary supplementation of *Perilla frutescens* seed meal on meat chemical composition in finishing castrated male Songliao black pigs

Items, %	CON	PFS3.0	PFS6.0	PFS9.0
Moisture	72.46 ± 0.55^a^	71.04 ± 1.02^b^	70.43 ± 0.79^b^	70.67 ± 1.24^b^
Crude protein	22.22 ± 0.55^b^	23.50 ± 0.71^a^	23.97 ± 0.35^a^	24.18 ± 0.79^a^
Crude fat	3.27 ± 0.20^b^	3.50 ± 0.30^a^ ^,^ ^b^	3.72 ± 0.13^a^	3.55 ± 0.14^a^ ^,^ ^b^
Ash	1.47 ± 0.27	1.96 ± 0.39	1.88 ± 0.49	1.70 ± 0.28

Abbreviations: CON, basal diet; PFS3.0, CON + 3% of *Perilla frutescens* seed; PFS6.0, CON + 6% of *Perilla frutescens* seed; PFS9.0, CON + 9% of *Perilla frutescens* seed.

^a,b^ Means in the same row with different superscripts differ (*p* < 0.05).

### 3.3 Meat free amino acids composition

The results of dietary supplementation of PFS on free AA in finishing male Songliao black pigs are shown in Table 6. The meat from the CON had significantly lower of aspartic acid, glycine, glutamic acid, alanine, serine, methionine, leucine, lysine, arginine, histidine, free AA, NEAA, EAA and total AA than the meat from the three levels of PFM treatments. Moreover, samples in the PFS9.0 group had significantly higher AA levels of aspartic acid and glycine compared to those fed the CON and PFM6.0 diets.

**TABLE 6:** Effects of dietary supplementation of *Perilla frutescens* seed on meat free amino acids composition in finishing castrated male Songliao black pigs

Amino acids, %	CON	PFS3.0	PFS6.0	PFS9.0
Aspartic acid	6.99 ± 0.24^c^	7.81 ± 0.33^a^ ^,^ ^b^	7.66 ± 0.38^b^	8.37 ± 0.43^a^
Glycine	2.69 ± 0.04^c^	3.01 ± 0.08^a^ ^,^ ^b^	3.07 ± 0.11^b^	3.43 ± 0.18^a^
Glutamic acid	11.75 ± 0.33^b^	13.27 ± 0.41^a^	13.31 ± 0.11^a^	13.66 ± 0.32^a^
Alanine	3.90 ± 0.21^b^	4.31 ± 0.16^a^	4.33 ± 0.06^a^	4.62 ± 0.23^a^
Cysteine	0.48 ± 0.03	0.47 ± 0.04	0.47 ± 0.04	0.49 ± 0.03
Proline	2.38 ± 0.17	2.39 ± 0.14	2.35 ± 0.09	2.37 ± 0.07
Tyrosine	2.33 ± 0.20	3.01 ± 0.12	2.96 ± 0.03	3.04 ± 0.11
Serine	3.01 ± 0.09^c^	3.19 ± 0.11^b^	3.13 ± 0.09^b^	3.45 ± 0.08^a^
Threonine	3.40 ± 0.12	3.75 ± 0.15	3.68 ± 0.14	3.51 ± 0.35
Methionine	1.02 ± 0.06^b^	1.79 ± 0.07^a^	1.81 ± 0.06^a^	1.75 ± 0.08^a^
Isoleucine	2.91 ± 0.28	2.96 ± 0.15	3.08 ± 0.06	3.03 ± 0.23
Leucine	6.29 ± 0.27^b^	6.66 ± 0.24^a^	6.73 ± 0.02^a^	6.58 ± 0.14^a^
Phenylalanine	2.64 ± 0.12	2.67 ± 0.09	2.67 ± 0.05	2.72 ± 0.11
Lysine	6.72 ± 0.25^b^	7.39 ± 0.33^a^	7.40 ± 0.10^a^	7.63 ± 0.11^a^
Arginine	4.35 ± 0.14^b^	4.74 ± 0.14^a^	4.84 ± 0.07^a^	4.88 ± 0.13^a^
Histidine	3.13 ± 0.10^b^	3.37 ± 0.11^a^	3.38 ± 0.13^a^	3.48 ± 0.11^a^
Valine	3.40 ± 0.22	3.50 ± 0.09	3.51 ± 0.05	3.56 ± 0.16
Free amino acid	25.36 ± 0.89^b^	28.47 ± 1.13^a^	28.43 ± 0.46^a^	30.12 ± 1.27^a^
NEAA	33.49 ± 0.72^b^	37.51 ± 1.52^a^	37.38 ± 0.44^a^	39.46 ± 1.32^a^
EAA	33.73 ± 1.43^b^	36.88 ± 1.40^a^	37.16 ± 0.14^a^	37.24 ± 0.95^a^
Total amino acid	67.21 ± 1.99^b^	74.39 ± 2.91^a^	74.54 ± 0.57^a^	76.70 ± 1.66^a^

Abbreviations: CON, basal diet; EAA, essential amino acid; NEAA, nonessential amino acid.; PFS3.0, CON + 3% of *Perilla frutescens* seed; PFS6.0, CON + 6% of *Perilla frutescens* seed; PFS9.0, CON + 9% of *Perilla frutescens* seed.

^a,b,c^ Means in the same row with different superscripts differ (*p* < 0.05).

### 3.4 Meat fatty acids composition

As shown in Table 7, dietary addition of PFS significantly affected the FA composition in Songliao black pigs. Pigs fed the PFS diets had higher levels of alpha linolenic acid, docosapentaenoic acid, eicosapentaenoic acid and docosahexaenoic acid in the meat compared with those fed the CON diet. Dietary supplementation of PFS significantly decreased SFA concentration. Following the addition of PFS, meat ω‐3 composition was increased.

**TABLE 7:** Effects of dietary supplementation of *Perilla frutescens* seed on meat fatty acids composition in finishing castrated male Songliao black pigs

Fatty acids, %	CON	PFS3.0	PFS6.0	PFS9.0
C4:0	9.57 ± 0.56	9.01 ± 1.20	9.16 ± 0.79	9.12 ± 0.96
C6:0	5.83 ± 0.50	6.20 ± 0.60	6.80 ± 0.64	6.48 ± 0.47
C8:0	0.03 ± 0.009	0.02 ± 0.005	0.02 ± 0.009	0.03 ± 0.008
C10:0	0.05 ± 0.007	0.05 ± 0.007	0.05 ± 0.008	0.05 ± 0.008
C12:0	0.06 ± 0.009	0.05 ± 0.008	0.05 ± 0.011	0.05 ± 0.010
C14:0	1.03 ± 0.06	0.98 ± 0.12	0.96 ± 0.23	0.93 ± 0.08
C16:0	20.94 ± 0.62^a^	20.15 ± 0.66^a^	18.36 ± 1.21^b^	18.59 ± 1.58^b^
C17:0	0.12 ± 0.01	0.13 ± 0.02	0.11 ± 0.02	0.12 ± 0.01
C18:0	9.06 ± 0.30^a^	8.70 ± 0.73^a^	8.31 ± 0.69^b^	8.26 ± 0.30^b^
C20:0	0.16 ± 0.01	0.14 ± 0.02	0.15 ± 0.02	0.15 ± 0.02
C21:0	0.05 ± 0.005	0.04 ± 0.005	0.05 ± 0.013	0.04 ± 0.011
C22:0	0.03 ± 0.011	0.04 ± 0.013	0.04 ± 0.008	0.03 ± 0.013
C23:0	0.71 ± 0.09^a^	0.65 ± 0.08^a^ ^,^ ^b^	0.48 ± 0.07^c^	0.56 ± 0.04^b^ ^,^ ^c^
C14:1	0.02 ± 0.007	0.02 ± 0.008	0.02 ± 0.011	0.03 ± 0.015
C16:1	2.86 ± 0.07	2.60 ± 0.34	2.72 ± 0.53	2.68 ± 0.30
C17:1	0.11 ± 0.02^a^	0.11 ± 0.02^a^	0.08 ± 0.02^b^	0.08 ± 0.02^b^
C20:1	0.65 ± 0.05	0.58 ± 0.05	0.59 ± 0.08	0.57 ± 0.08
C24:1	0.11 ± 0.015^a^	0.08 ± 0.011^b^	0.06 ± 0.011^b^	0.06 ± 0.004^b^
C18:1n9t	39.19 ± 0.51	39.19 ± 1.49	39.48 ± 1.18	39.60 ± 1.26
C18:1n9c	3.82 ± 0.14	3.73 ± 0.22	3.79 ± 0.14	3.72 ± 0.40
C22:1n9	0.02 ± 0.007	0.02 ± 0.008	0.03 ± 0.011	0.02 ± 0.013
C20:2	0.17 ± 0.01	0.18 ± 0.02	0.17 ± 0.02	0.17 ± 0.02
C22:2	0.03 ± 0.011	0.03 ± 0.013	0.04 ± 0.017	0.04 ± 0.015
C18:2n6c	4.76 ± 0.74	5.77 ± 0.57	5.73 ± 0.69	5.78 ± 0.97
C18:2n6t	0.15 ± 0.02	0.13 ± 0.01	0.16 ± 0.03	0.14 ± 0.02
C18:3n6	0.21 ± 0.03^a^	0.04 ± 0.01^b^	0.04 ± 0.01^b^	0.04 ± 0.01^b^
C20:3n6	0.10 ± 0.01	0.12 ± 0.02	0.12 ± 0.02	0.10 ± 0.01
C18:3n3(ALA)	0.03 ± 0.01^c^	0.74 ± 0.05^b^	1.77 ± 0.13^a^	1.79 ± 0.10^a^
C20:3n3(DPA)	0.04 ± 0.01^c^	0.13 ± 0.02^b^	0.27 ± 0.05^a^	0.30 ± 0.03^a^
C20:5(EPA)	0.03 ± 0.01^d^	0.14 ± 0.03^c^	0.17 ± 0.01^b^	0.22 ± 0.02^a^
C22:6 ns(DHA)	0.08 ± 0.01^b^	0.23 ± 0.02^a^	0.23 ± 0.03^a^	0.24 ± 0.03^a^
SFA	47.63 ± 0.38^a^	46.16 ± 1.27^a^ ^,^ ^b^	44.54 ± 1.29^b^	44.41 ± 2.09^b^
MUFA	46.78 ± 0.54	46.33 ± 1.58	46.77 ± 1.65	46.76 ± 1.40
PUFA	5.59 ± 0.71^c^	7.51 ± 0.66^a^ ^,^ ^b^	8.69 ± 0.63^a^	8.83 ± 0.92^a^
MUFA+PUFA	52.37 ± 0.38^b^	53.84 ± 1.27^a^ ^,^ ^b^	55.46 ± 1.29^a^	55.59 ± 2.09^a^
ω‐6	5.22 ± 0.71	6.06 ± 0.59	6.05 ± 0.67	6.07 ± 0.96
ω‐3	0.17 ± 0.03^c^	1.24 ± 0.07^b^	2.44 ± 0.16^a^	2.55 ± 0.11^a^

Abbreviations: ALA, alpha linolenic acid; CON, basal diet; DHA, docosahexaenoic acid; DPA, docosapentaenoic acid; EPA, eicosapentaenoic acid; MUFA, monounsaturated fatty acid; PFS3.0, CON + 3% of *Perilla frutescens* seed meal; PFS6.0, CON + 6% of *Perilla frutescens* seed meal; PFS9.0, CON + 9% of *Perilla frutescens* seed meal; PUFA, polyunsaturated fatty acid; SFA, saturated fatty acid.

^a,b,c^ Means in the same row with different superscripts differ (*p* < 0.05).

## 4 DISCUSSION

Pork is a primary meat product for consumers worldwide. Data indicate that global pork production for 2021 is approximately 103.8 million tons, even though the Chinese hog sector is still affected by xinAfrican Swine fever (https://apps.fas.usda.gov/psdonline/circulars/livestock_poultry.pdf). Furthermore, strict human food safety and health requirements request that producers not only improve the quality but also on the flavour and nutrition of pork. The Songliao black pig is a local swine that is well known for its meat quality (Hui, 2017). To obtain better flavour, the aim of this study was to examination the effects of PFS on growth performance and blood profiles, particularly on meat quality, and meat nutritional parameters in finishing castrated male Songliao black pigs. The results of this experiment showed that the PFS9.0 observed lowest FBW, ADFI and ADG compared with other treatments. On the contrary of this experiment, Pan (2012) reported that dietary supplementation of 200 mg/kg PF extract improved ADG by 5.63% and decreased FCR by 7.59% in crossbreed finishing pigs. In line with Pan's research, Hu et al. (2011) also indicated that a 350 g/ton addition of PF extract increased ADG and reduced FCR in finishing pigs; moreover, it was also shown that supplementation increased carcass yield by nearly 2%. A positive influence has also been seen in other pigs. For instance, Zhou et al. (2019) reported that inclusion of 250–400 g/ton PF extract in weaning pig diet improved growth performance. Recently, Zhang et al. (2017) showed that dietary supplementation of 1000 mg/kg of PF extract significantly decreased FCR and increased the blood concentration of IgG in Congjiang piglets. As a medicinal and food homologous plant, PFS is reported to have a variety of natural molecules such as protein, essential oils, phenolic acids and flavonoids (Peng et al., 2005). Based on these biological ingredients, PFS has been shown to have antioxidant, antimicrobial and anti‐inflammatory activity (Peng et al., 2018). The influence on growth performance may be due to improvement of the pigs’ health through PFS supplementation. Moreover, the decreased performance in PFS9.0 group may be because of the higher PFS supplementation increased the dietary fibre content which negatively influenced growth performance. Further studies are still needed on the effects of PFS on pigs.

Data of the influence of dietary supplementation of PFS on blood profiles in pigs is limited, and most studies have focused on immune function parameters. Thus, the direct comparisons could not be made on the blood profiles in PFS in Songliao black pigs. Albumin is a kind of major serum protein in the mammalian blood that plays a role in maintaining nutrition and blood osmotic pressure (He & Carter, 1993). BUN is considered as an accurate parameter to reflect protein metabolism and the balance of amino acids (Sun et al., 2019). In addition, the triacylglycerol, cholesterol and LDL‐C are well‐known lipid indexes in the blood. Results showed that dietary addition of PFS influenced blood protein and lipid parameters in castrated male Songliao black pigs in this experiment. It has been reported that dietary supplementation of PFS extracts decreased blood BUN concentration (Zhang et al., 2017). Other studies have shown that supplementation with PFS products improved immune parameters in pigs (Cai et al., 2017). Zhu et al. (2020) also reported that dietary inclusion of PFS protein positively influenced immune regulation in immunocompromised mice. Furthermore, lower triacylglycerol, cholesterol and LDL‐C levels are recognised as beneficial to human and animal health (Sun et al., 2020). In agreement with this study, Liu and Kim (2018) also found that dietary replacement of soybean oil with linseed oil significantly reduced serum cholesterol, triglycerides and LDL‐C concentrations in crossbreed finishing pigs. PFS is a good FA source that is rich in palmitic acid, stearic acid, oleic acid, linoleic acid and linolenic acid. PFS contains a high level of α‐linolenic acid (C18:3 n‐3; ALA), which ranges from 52% to 62% (Ahmed, 2018). The changes in blood profiles in this experiment may be due to the FAs from PFS.

In the current study, dietary supplementation of PFS significantly influenced meat characteristics such as colour, pH, pressing loss, cook meat rate, moisture, crude protein, crude fat, AA composition and FA composition. The results suggest that the addition of PFS had a positive effect on meat quality and nutritional parameters in finishing castrated male Songliao black pigs. Consistent with this study, Zhao (2018) reported that dietary PFS extract supplementation improved meat colour, lean meat percentage, drip loss and intramuscular fat in crossbred finishing pigs. Similarly, Wang and Sun (2018) also found that dietary addition of complex plant extracts including PFS increased the meat colour score and lean meat percentage and decreased the backfat, which suggested positive impacts on the carcass and meat quality. Studies focused on the application of PFS in monogastric animal are limited. Nevertheless, studies have reported that PFS has the ability to influence the product quality, specially the FA and AA composition of livestock products (Peng et al., 2018). This may explain the improvement in meat quality and nutritional parameters in this study.

AAs are basic units that play an important role in mammalian animal protein make up. Additionally, AAs can be divided into three types of tastes, namely, sweet, bitter and umami which are related to the taste of the meat (Vieillevoye et al., 2020; Gao et al., 2018). The results of this study show that dietary PFS altered meat AA composition; in particular, aspartic acid and glutamic acid were increased. Moreover, the composition of glycine, methionine, arginine, leucine and alanine were also increased in this study. Based on their classification, glycine, methionine, arginine, leucine and alanine are considered sweet AAs, aspartic acid and glutamic acid which are well known as the umami AAs (Guo et al., 2019). It has been suggested that sweet AAs help to enhance umami taste and that umami AAs also have a synergistic effect with taste nucleotides that contribute to increasing the umami taste (Tu et al., 2020). PFS was reported to have a high protein content (20–30%) with eight types of AAs (Jia et al., 2016). Promotion of meat AA composition in this experiment may be due to the high content and variety of AAs in PFS.

This experiment also showed changes in meat FA composition of castrated male Songliao black pigs. It has been reported that ω‐3 FAs play an important role in the inflammatory response, reproductive performance and meat nutritional characteristics in pigs (Sun et al., 2020). Furthermore, for health reasons, the consumers prefer products with a higher ω‐3 FA content or lower ω‐6 to ω‐3 ratio. Previous studies focused on altering dietary FA ratios to influence the meat quality of finishing pigs. However, few of these studies tested the FA composition of meat. Wood et al. (2004) suggested that in monogastric animals, the lipid composition of meat might reflect the nature of dietary fats. Moreover, the technological and sensory parameters of pork have also been reported to be affected by FA profiles (Rossi et al., 2010). The ALA content in PFS has been reported at a relatively high level from 39% to 73% (Jia et al., 2016). Therefore, the improvement of meat quality and nutritional parameters may also be due to changes in meat FA composition. In addition, the lipid oxidation is another factor that could affect the meat quality and meat composition (Cannata et al., 2010). Due to a lack of literature, direct comparisons of PFS on meat FA composition in pigs are impossible. However, we may conclude that dietary supplementation of PFS in Songliao black pigs had no significant deleterious effects on meat FA composition relevant to product safety.

Dietary supplementation of PFS in a finishing castrated male Songliao black pig diet improved growth performance, blood profiles, meat quality and meat nutritional parameters. PFS enhanced the meat AA composition and FA composition, which may result in a better tasting and healthier meat. This study provides new evidence and insight for the use of PFS in a native breed of pigs to obtain higher quality meat products.

## FUNDING INFORMATION

National Natural Science Foundation of China:31872980 and U20A2052; China Agricultural Research System: CARS‐37.

## CONFLICT OF INTEREST

We wish to confirm that there were no conflicts of interest associated with the publication.

## AUTHOR CONTRIBUTIONS

Ji Qiao Xia: data curation; formal analysis; methodology; software; validation; visualisation; writing – original draft; writing – review & editing. Xinmiao He: data curation; methodology; software; visualisation. Lan Wang: formal analysis; methodology; software; visualisation. Liang Wang: data curation; formal analysis; methodology; software; visualisation. Dong Ji Zhang: data curation; formal analysis; methodology; software; visualisation. Ji Feng Wang: data curation; formal analysis; methodology; software; visualisation. Di Liu: conceptualisation; project administration; resources; supervision.

## ETHICS STATEMENT

The authors confirm that the ethical policies of the journal, as noted on the journal's author guidelines page, have been adhered to and the appropriate ethical review committee approval has been received. The authors confirm that they have followed EU standards for the protection of animals used for scientific purposes [and feed legislation, if appropriate. The experimental protocol used in this study was approved by the Animal Care and Use Committee of Northeast Agricultural University, People's Republic of China.

## Data Availability

The datasets produced and/or analyzed during the current study are available from the corresponding author on reasonable request.
